# Estimation of Redox Status in Military Pilots during Hypoxic Flight-Simulation Conditions—A Pilot Study

**DOI:** 10.3390/antiox11071241

**Published:** 2022-06-24

**Authors:** Konstantina Petraki, Maria G. Grammatikopoulou, Fotios Tekos, Zoi Skaperda, Marina Orfanou, Robin Mesnage, Tonia Vassilakou, Demetrios Kouretas

**Affiliations:** 1 Department of Public Health Policy, School of Public Health, University of West Attica, Athens University Campus, GR-11521 Athens, Greece; ntinapetraki@hotmail.com; 2 251 General Airforce Hospital, GR-15561 Athina, Greece; 3 Department of Nutritional Sciences & Dietetics, Faculty of Health Sciences, International Hellenic University, Alexander Campus, GR-57400 Thessaloniki, Greece; mariagram@auth.gr; 4 Department of Rheumatology and Clinical Immunology, Faculty of Medicine, School of Health Sciences, University of Thessaly, University General Hospital of Larissa, GR-41110 Larissa, Greece; 5 Department of Biochemistry and Biotechnology, University of Thessaly, Viopolis, Mezourlo, 41500 Larissa, Greece; ftekos@uth.gr (F.T.); zoskaper@bio.uth.gr (Z.S.); marinaorfanou13@gmail.com (M.O.); 6 Gene Expression and Therapy Group, Department of Medical and Molecular Genetics, Faculty of Life Sciences & Medicine, King’s College London, 8th Floor, Tower Wing, Guy’s Hospital, Great Maze Pond, London SE1 9RT, UK; robin.mesnage@kcl.ac.uk; 7 Buchinger Wilhelmi Clinic, Wilhelmi-Beck-Straße 27, 88662 Überlingen, Germany

**Keywords:** oxidative stress, aviation, aerospace medicine, hypoxia, hypoxia-inducible factor, airforce

## Abstract

At high altitude conditions, the low-pressure atmospheric oxygen reduces the generation of energy, thus inducing a decrease in oxygen availability. As a result, endurance flights evoke imbalance in redox signaling, posing a safety risk for the pilots involved. The aim of the present study was to assess changes in the redox status of military pilots during flight simulation conditions according to their flight hours (experts vs. novice). A total of seven expert pilots and an equal number of novice pilots (trainees) were recruited from the Center for Airforce Medicine of the Greek Military Airforce. Glutathione (GSH) levels, catalase activity (CAT), total antioxidant capacity (TAC), lipid peroxidation through the thiobarbituric acid-reactive substances (TBARS), and protein oxidative damage through the assay of protein carbonyls (PCs) levels were assessed at two time points, once prior to and once immediately post a scheduled flight simulation. In the experienced pilots’ arms, GSH was significantly increased post-flight simulation, with TAC being simultaneously reduced. On the other hand, in the trainees’ arms, CAT and TAC were both increased post-flight. No differences were noted with regard to the TBARS and PCs post-simulation. When the two groups were compared, TAC and PCs were significantly lower in the trainees compared to the experienced pilots. The present study provides useful insight into the physiological redox status adaptations to hypobaric hypoxic flight conditions among pilots. In a further detail, an increase in GSH response post-flight simulation is being evoked in more experienced pilots, indicating an adaptation to the extreme flight conditions, as they battle oxidative stress.

## 1. Introduction

Aerospace medicine is a sub-specialty of the discipline of Occupational Medicine, focusing on ensuring the safety, health and performance of pilots and personnel exposed to air- and space-operational settings [1]. Among the unique challenges of the fighter cockpit environments posing additional physiological stress to the military pilots, are exposure to radiation, microgravity or hypoxic conditions, multi-axial G-forces, and elevated temperature and humidity, etc. [1,2,3].

More importantly, at high altitude, the low-pressure atmospheric oxygen reduces the generation of energy, thus inducing a decrease in oxygen availability [4]. This is accompanied by an increased formation of reactive oxygen and nitrogen species (RONS) and a greater degree of oxidative damage to the lipids, proteins and DNA, affecting cellular and organ function [5,6]. Redox status imbalance is apparent during hypobaric hypoxia and altitude, depleting the antioxidant system and its capacity to withstand RONS produced during exercise [7,8,9].

Evidence from the animal kingdom suggests that endurance flights evoke crucial threats for the antioxidant arsenal of cells. Several markers of oxidative damage, including protein carbonyls (PCs), malondialdehyde (MDA) and proxies of enzymatic antioxidant capacity including glutathione peroxidase (GPx), appear to be affected during bird migration flights [10,11,12,13].

On the other hand, conflicting evidence is apparent in humans. Research on Italian supersonic aircraft pilots failed to indicate differences in the redox status biomarkers compared to healthy, non-pilot controls [14]. Similarly, healthy Polish pilots exhibited SOD, GPx and TAC within the normal ranges while executing two training flights and participating in a human centrifuge test [15]. On the other hand, when Iranian helicopter pilots were compared to non-flight staff [16], greater concentrations of erythrocytes superoxide dismutase (SOD), serum MDA, total antioxidant capacity (TAC) and erythrocytes GPx were noted in the pilots compared to the non-flight staff. Comparison of the production of free radicals and antioxidant defenses among Russian cosmonauts, airline pilots, train engine drivers and age-matched controls indicated higher granulocyte superoxide and nitric oxide levels, elevated erythrocyte SOD activity and glutathione (GSH) oxidation in the cosmonauts, compared to the rest of the groups [17].

These differences in the results might well be coincidental. Furthermore, it is highly possible that adaptive mechanisms are developed progressively, protecting pilots from excessive oxidative damage. Thus, it could be argued, that novice and well-experienced pilots might demonstrate variation in the critical endpoints of redox signaling. In this context, the aim of the present quasi-experimental study was to evaluate changes in the redox status of military pilots during flight simulation conditions according to their flight hours (expert vs. novice).

## 2. Materials and Methods

### 2.1. Participants

Apparently healthy pilots were recruited from the Center for Airforce Medicine of the Greek Military Airforce. A total of seven expert pilots and an equal number of novice pilots (trainees) were recruited, forming two groups, respectively. The characteristics of participants are reported in Table 1.

Exclusion criteria involved any history of musculoskeletal injury and not providing consent to participate in the study. Inclusion criteria involved all expert and trainee pilots agreeing to participate in the study. During the study period, all participants were advised to abstain from using anti-inflammatory or analgesic medication.

### 2.2. Study Ethics

Permission for the study was provided by the 251st General Airforce Hospital and the Director of the Center for Airforce Medicine of the Greek Military Airforce. All participants provided informed consent prior to participation.

### 2.3. Anthropometric Measurements

Body weight of participants was measured by an experienced dietitian with the use of a digital scale (Tanita 780, Tanita, Amsterdam, The Netherlands), with all pilots being barefoot and undressed. Height was measured with a portable stadiometer (Tanita HR 001, Tanita, Amsterdam, The Netherlands). Body mass index (BMI) was calculated for all participants.

### 2.4. Procedures and Flight Simulation Protocol

All measurements were taken at two time points, once prior to and once immediately post a scheduled flight simulation. The capacity of the flight simulation (hyperbaric) chamber involved 5 pilots in total.

Each flight was preceded by a de-nitrogenation period, through the supply of oxygen (100%) for half an hour, aiming to prevent dysbarism. Then the flight was initiated, at a speed of 2500 ft/min reaching 5000 ft, followed by the descent at the same pace, until 1000 ft of height were reached. This part of the flight aims to familiarize participants with the Valsalva maneuver [18]. Thereafter, an ascent at a speed of 2500 ft/min until reaching 25.000 ft of altitude was performed, and the acute hypoxia test took place. At this point, participants were asked to take off their masks and remain without oxygen for approximately 2–3 min, until they felt symptoms of hypoxia, which were recorded. Oxygen saturation was constantly recorded for each participant through oximeters and, during this part of the flight, oxygen saturation reached 60–65%. Immediately after the symptoms of hypoxia were reported, participants were allowed to put their masks back on, balancing oxygen saturation once again within the normal ranges. In the cases where participating pilots had lost their senses, a trained physician accompanying each simulation flight was responsible for putting the mask back on to every affected pilot.

Once the test flight was completed by all participants, the descent began at the same speed. This speed, however, could be slowed further, in cases of any inconvenience reported by the participating pilots. Approximately half an hour after the exposure to hypoxia, participants were released from the chamber.

The rate of ascent used in the flight simulation chambers was approximately 6 times greater compared to that of the military airplanes.

### 2.5. Blood Collection

Blood samples were collected twice, before and after the flight simulation, by experienced medical-technical assistants between 7.30–9.00 a.m. A total of 10 mL of blood were drawn from the forearm vein, with the pilots being seated in an upright position. Blood was collected in ethylenediaminetetraacetic acid (EDTA) tubes, centrifuged immediately (1370× *g* for 10 min at 4 °C), and the plasma was isolated and placed in Eppendorf tubes. The remaining packed erythrocytes were lysed with dH_2_O (1:1 *v*/*v*), inverted vigorously and centrifuged (4020× *g* for 15 min at 4 °C). The erythrocyte lysate was collected in additional Eppendorf tubes. Both plasma and erythrocyte lysate samples were stored at −80 °C, until the biochemical analyses were performed.

### 2.6. Redox Status Assays

The redox status of pilots was assessed through five distinct redox biomarkers in plasma and red blood cells. Blood samples were analyzed in the laboratory of Animal Physiology at the Department of Biochemistry and Biotechnology, University of Thessaly. Five samples were taken and analyzed for each participant, at each time point and for each marker, in order to increase the validity of the findings. The assessment of GSH levels, catalase activity (CAT), TAC, lipid peroxidation through the thiobarbituric acid-reactive substances (TBARS), and protein oxidative damage through the assay of PCs levels were performed, as already described in previous studies [19,20].

In a further detail, for the determination of TBARS in plasma, a slightly modified assay of Keles et al. [21] was applied. PCs were assessed according to Michailidis et al. [22]. For CAT activity in erythrocytes, the method of Aebi was used [23]. GSH levels in erythrocytes were assessed using the Reddy et al. method, as previously described by Veskoukis and associates [24]. The determination of TAC was based on the method of Janaszewska and Bartosz [25] and were measured in plasma as mmol of 2,2-diphenyl-1-picrylhydrazyl (DPPH) reduced to 2,2-diphenyl-1-picrylhydrazine (DPPH:H).

### 2.7. Statistical Analyses

All analyses were performed using the Statistical Program for Social Sciences (SPSS, SPSS Inc., Chicago, IL, USA) and the level of significance was set at 0.05%. Data are presented as means with their respective standard deviations (SDs). Markers of oxidative stress were compared using one-way ANOVA analysis. Before-after comparisons were conducted using the Dunnett’s test.

## 3. Results

The expert pilots were significantly older than the novice group (*p* ≤ 0.001). The rest of the characteristics do not reveal any significant alteration between the study groups. Table 2 details the results regarding the redox status of participants before and immediately after the flight simulation, per group. The results suggest that, in the experienced pilots’ arms, GSH was significantly increased post-flight simulation, with TAC being simultaneously reduced. On the other hand, in the trainees’ group, CAT and TAC were both increased post-flight. No differences were noted with regard to the TBARS and PCs post-simulation.

**Figure 1:**
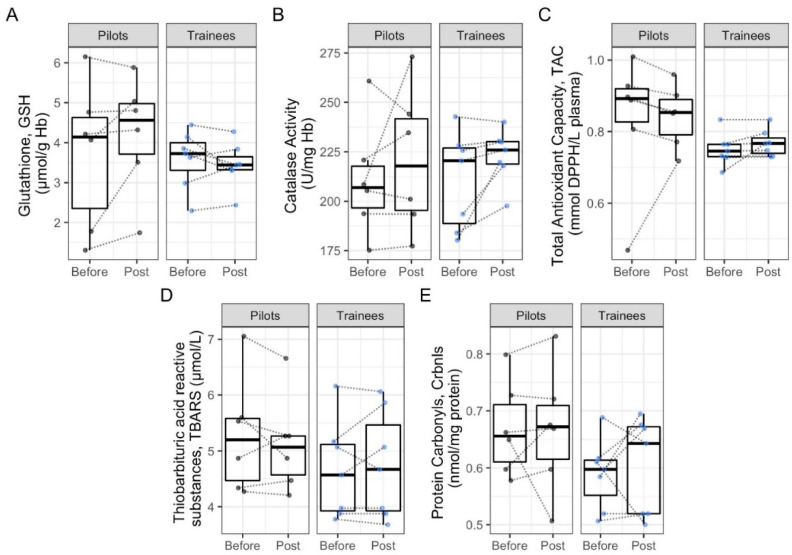
Results of the redox status markers of participants before and after the flight simulation. (**A**) Glutathione, GSH; (**B**) Catalase Activity; (**C**) Total Antioxidant Capacity, TAC; (**D**) Triobarbituric acid reactive; (**E**) Protein Carbonyls, Crbnls.

When the two study groups were compared, TAC and PCs were significantly lower in the trainees compared to the experienced pilots both before and post simulation (Table 2, Figure 1). No other differences were noted.

## 4. Discussion

The present study assessed the effects of a flight simulation on the oxidative modifications of proteins and lipids through the PCs and TBARS assays, respectively [26], as well as the antioxidant status of the pilots, through the assessment of TAC, CAT and GSH [27,28]. The results revealed that inexperienced pilots exhibited lower TAC and PCs compared to the more experienced ones, both before and post simulation. Furthermore, hypoxic flight simulation conditions induced different results in the redox balance of participants in the two groups, with CAT and TAC being increased in the novice pilots’ arms and GSH being significantly elevated in the experienced pilots group.

Environmentally induced hypoxemia can alter the balance between antioxidant defense mechanisms and the production of reactive species, propelling oxidative damage [29,30]. However, it should be noted that, apart from the environment, redox status is greatly affected by age. TAC in particular is reduced with ascending age [31] and this might partly explain the elevated TAC of the trainee pilots post-simulation compared to before, as they were younger than the expert pilots; therefore, they were capable of counteracting oxidative stress directly. On the other hand, protein carbonylation is considered to be a pivotal indicator of protein-oxidative stress and one of the hallmarks of cellular aging, with greater PCs levels being apparent in older individuals [32,33,34]. For this, between groups, PCs concentrations were elevated in the expert pilots compared to the younger and less experienced ones. In previous research, Dogliotti [14] compared the PCs levels of young supersonic pilots to that of middle-aged, healthy controls and found similar results between groups, suggesting a protective effect against frequent exposure to high altitude. However, the sample used by Dogliotti and associates [14] included pilots of similar age to the trainees included herein, whereas the healthy controls were middle-aged men, with an age similar to that of the expert pilots herein. Thus, one could argue that if the groups were more comparable age-wise with older, more experienced pilots being employed in Dogliotti’s case-control study [14], PCs levels might have been different, and the possible effect of high altitude might have been more apparent, as seen in the present study. In vitro and animal studies revealed a sharp increase in PCs accumulation during the last third of the lifespan of various organs/species, including rat liver [35], house flies [36], or human cultured dermal fibroblasts [37], linking PCs content to life expectancy [38]. Similarly, research on humans has also demonstrated that saliva and plasma PCs content are significantly correlated with age, and their assay has been suggested as a proxy aging marker [32]. This expected increase in PCs concentrations might be the result of the synergic effect of chlorinative stress, advanced oxidation protein products (AOPP), protein *Tyr* nitration and many more mechanisms observed during aging [38]. Thus, the elevated PCs levels and the lower TAC post simulation compared to those before simulation that were observed in the expert pilots herein could be possibly attributed to aging, rather than an adaptive flight response.

The hypoxia-inducible factor (HIF) consists of the major oxygen sensor within the cell environment, central to the regulation of cell response to different oxygen levels [39,40]. During hypoxic conditions, HIF activation ensures the optimum production of ATP and is associated with the formation of reactive oxygen species (ROS) [41]. HIF activation can either lower the rate of ROS production through the suppression of the mitochondrial TCA cycle, or accelerate ROS production via NADPH oxidase (NOX) [42]. When pilots are not trained in hypoxic, high-altitude environments, HIF has a detrimental effect in the awareness of environment, cognition and decision making [43], as seen in helicopter pilots [44]. For this, adaptation mechanisms to tampering down ROS production are of pivotal importance for the safety of the flight crew. In this context, CAT and GSH are components of the antioxidant defense grid, suppressing the formation of free radicals or reactive species in cells [45,46,47]. CAT consists of a first-line antioxidant (enzymes), and GSH belongs at the second line of defense (ancillary factors) [45]. In the present study, TAC and CAT were increased only in the trainee group, and GSH was increased among expert pilots post-flight simulation. Thus, it could be hypothesized that different defense responses were triggered in each group, following the increased redox deregulation in these conditions. On the other hand, one could also argue that these differences are once again due to the age difference observed between participants, or the epiphenomenon of an adaptation following prolonged exposure to hypoxic conditions. Previous research on animals has suggested an age-related decrease in the GSH content of several tissues, paired with an increase in GSH oxidation to glutathione disulfide (GSSG) [48,49,50]. Thus, a decrease in GSH would have been expected in the older, expert pilots, whereas instead, an increment is noted post-flight simulation. Similarly, previous research also showed that adhering to a specific exercise schedule for more extended training periods improved GSH levels in humans, suggesting the development of a steady adaptation response [51,52] suppressing skeletal muscle fatigue [53]. On the other hand, according to a meta-analysis, TAC appears moderately increased in shorter training periods, as seen herein among the less experienced pilots [52]. In line with these findings, it was also demonstrated that Iranian pilots exhibited greater resting levels of GSH and TAC compared to non-flight staff [16]. Collectively, these results suggest that, during the early stages of the hypoxic pilot training, TAC is employed to rebalance redox status, whereas, during long-term periods of training in hypoxic conditions, GSH is adaptively increased, as seen in the expert pilots.

Another mechanism that might possibly explain the present findings lies in the “normobaric oxygen paradox” (NOP) [54], which involves the response when returning to normoxia after a hyperoxic event. During this return to normal oxygen levels, the tissues experience an oxygen shortage, up-regulating the HIF-1α transcription-factor activity [54]. According to Fratantonio [54], high and very high hyperoxia induce a progressive shift towards an oxidative stress response, overlapping to hypoxia [55], while elevating GSH and matrix metallopeptidase 9 levels.

### Limitations of the Study

Although the present study is biased by the small sample of recruited pilots, it consists of one of the first attempts to assess changes in the redox status of pilots’ pre- and post-flight simulations, according to their level of expertise. Nevertheless, the results cannot be used to generalize the findings, as pilot studies are often characterized by statistical uncertainty [56]. The absence of participants with similar ages of both sexes represents another factor that our study does not explore, thus not enabling us to eliminate limitations that usually arise concerning the diversity of the two study groups. Of note, it should also be stated that the level of physical activity, body composition (including adiposity) and diet may also impact redox status [57,58,59,60,61], but were not accounted for herein. The regulation of these parameters is dependent on many environmental or genetic factors which have not yet been elucidated, representing a promising field of investigation.

## 5. Conclusions

The present study provides useful insight on the physiological redox status adaptations to hypoxic hypobaric flight conditions among pilots. In further detail, an increase in GSH response post-flight simulation is being evoked in more experienced pilots, indicating an adaptation to the flight training in an effort to battle oxidative stress. The redox status during hypoxia due to flight conditions should be further investigated through larger-scale studies among pilots, as this may affect their future health. Future studies should include participants of a wider age range and both sexes, estimation of additional redox markers, like antioxidant enzymes, and more specific biomarkers. It would be of particular interest for future studies to examine if oral nutrient supplementation with antioxidants or GSH could further impact redox status in post-flight simulations and improve the physiological response of the pilots.

## Figures and Tables

**Table 1:** Characteristics of the sample.

	Pilots (*n* = 7)	Trainees (*n* = 7)	*p* Value
Age (years)	41.7 ± 3.1	19.6 ± 0.3	0.0004
Body weight (kg)	83.5 ± 6.2	75.3 ± 2.8	0.12
Height (cm)	177.5 ± 1.8	175.9 ± 2.0	0.27
BMI (kg/m^2^)	26.5 ± 1.9	24.3 ± 0.5	0.15

BMI: body mass index.

**Table 2:** Results of the redox status markers of participants before and after the flight simulation.

	Pilots (*n* = 7)		Trainees (*n* = 7)	
	Before Simulation	Post-Simulation	Before Simulation	Post-Simulation
GSH (μmol/g of Hb)	3.73 ± 1.81	4.22 ± 1.43 *	3.5 ± 0.78	3.4 ± 0.61
CAT (U/mg of Hb)	210 ± 29	220 ± 36	210 ± 24	223 ± 13 *
TAC (mmol DPPH/L of plasma)	0.87 ± 0.12	0.84 ± 0.08 *	0.75 ± 0.04 ^†^	0.77 ± 0.03 *^,†^
TBARS (μmol/L)	5.28 ± 1.03	5.12 ± 1.86	4.66 ± 0.84	4.74 ± 0.96
PCs (nmol/mg of protein)	0.67 ± 0.08	0.67 ± 0.11	0.59 ± 0.06 ^†^	0.6 ± 0.08

CAT: catalase activity; DPPH: 2,2-diphenyl-1-picrylhydrazyl; GSH: glutathione; Hb: haemoglobin; PCs: protein carbonyls; TAC: total antioxidant capacity; TBARS: Thiobarbituric acid-reactive substances. * Statistically different compared to the baseline results of the same group according to the Dunnett’s test (*p* < 0.05); ^†^ Statistically different compared to the results of the opposite group, at the same timepoint, based on the ANOVA (*p* < 0.05).

## Data Availability

Data is contained within the article.
